# Energetic Costs of Extreme Heat: High Temperatures Elevate Daytime Activity and Suppress Nighttime Foraging in Flying‐Foxes

**DOI:** 10.1002/ece3.73408

**Published:** 2026-04-08

**Authors:** Melissa J. Walker, Justin A. Welbergen, Jessica Meade, Wayne S. J. Boardman, John M. Martin, Christopher Turbill

**Affiliations:** ^1^ Hawkesbury Institute for the Environment Western Sydney University Richmond New South Wales Australia; ^2^ School of Life and Environmental Sciences, Faculty of Science, Engineering and Built Environment Deakin University Burwood Victoria Australia; ^3^ Department of Pathobiology and Population Health, School of Animal and Veterinary Sciences University of Adelaide Roseworthy South Australia Australia; ^4^ Conservation and Ecology Institute Ecosure Brisbane Queensland Australia; ^5^ School of Science Western Sydney University Richmond New South Wales Australia

**Keywords:** acceleration, bats, climate change, energy expenditure, mammal, *Pteropus*, thermoregulation

## Abstract

Extreme heat weather events can lead to energy deficits by increasing thermoregulatory energy costs while reducing energy intake because of limits to dissipating the metabolic heat generated by foraging activity. Understanding the consequences of exposure to extreme heat is increasingly important under ongoing climate warming. We measured acceleration forces using collar‐mounted loggers to gain indices of activity and energy expenditure in grey‐headed flying‐foxes ( *Pteropus poliocephalus* ) during mild and hot summer conditions. Overall dynamic body acceleration (ODBA) is positively correlated with energy expenditure in a wide range of animals. During the daytime rest phase, air temperatures above 30°C decreased low‐level activity indicative of rest, whereas during the nocturnal active phase, temperatures above 21°C reduced high‐level activity indicative of flight. On hot days (maximum *T*_*a*_: 40.6°C ± 3.1°C), ODBA increased threefold during the daytime and decreased by one‐third during the first 2 h of the night, compared to milder summer conditions. These patterns indicate that extreme heat simultaneously elevates energy expenditure and constrains foraging activity. Our data support the hypothesis that heat‐induced energetic deficits are an important component of the negative effects of extreme heat exposure. Such energy deficits could reduce survival and population growth, especially as heat events become more frequent and severe under climate change.

## Graphical abstract

Extreme heat can create an energy deficit in animals by increasing thermoregulatory costs while limiting foraging activity, making its impacts increasingly important under global warming. Using collar‐mounted accelerometers, we quantified activity and energy expenditure in grey‐headed flying‐foxes across mild and hot summer conditions, finding that high temperatures increase rest‐phase daytime activity but reduce active‐phase nighttime flight. These actitivy shifts indicate a heat‐related energetic deficit that may negatively impact population growth and survival as extreme heat events become more frequent.

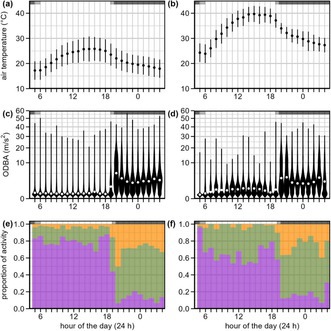

## 1 Introduction

Extreme heat can exceed the thermoregulatory capacities of birds and mammals and directly result in mortality, sometimes at a massive scale (e.g., Mo et al. 2022; Welbergen et al. 2008). In addition, the physiological and behavioural responses used by endothermic animals can have important non‐lethal consequences (Stiegler et al. 2023), including on individual energetic state, which can reduce individual fitness (Sharpe et al. 2019) and population growth rates (Wingfield et al. 2017; Fuller et al. 2021). The frequency, intensity and geographic extent of extreme heat events are increasing with climate change (Stillman 2019), and these events are predicted to occur more often in the future (Steffen et al. 2014), threatening the viability of many animal populations (McKechnie and Wolf 2010; Murali et al. 2023). The increasing threat of extreme heat events makes it a priority to understand the mechanisms by which these events negatively affect individuals, thereby informing mitigation and conservation strategies.

Animals use behavioural and physiological mechanisms to maintain body temperature within survivable limits. They may move to cooler microclimates (e.g., shade) or employ behaviours that provide a cooling effect (e.g., avoidance of radiative heat sources or immersion in water; Huey et al. 2012). A common physiological response to hot conditions, especially for animals in more arid habitats, is a controlled increase in body temperature. This response helps maintain a thermal gradient between the body surface and the environment that is necessary for non‐evaporative heat loss (Schmidt‐Nielsen et al. 1956; Gerson et al. 2019; Walker et al. 2025). Heat loss is enhanced by peripheral vasodilation and convection of air across body parts with large surface areas (e.g., ears, wings; Fuller et al. 2016; Smit et al. 2016). However, as air temperature approaches and exceeds an upper limit of body temperature, evaporative cooling becomes the only effective heat‐dissipation mechanism. Evaporative water loss is enhanced by panting, sweating, salivating and licking (McKechnie and Wolf 2019), and these active mechanisms increase resting metabolic rate (Fuller et al. 2016; McKechnie et al. 2021), thereby adding to an animal's overall thermal load and energetic costs.

Extreme heat increases thermoregulatory energy costs and can also restrict activity during hot conditions, particularly vigorous movements such as flying in birds or bats (Carpenter 1985). Extreme heat events have been associated with reduced foraging activity or efficiency and consequential decreases in body condition and reproductive success in bird and mammal populations (Woodroffe et al. 2017; Cunningham et al. 2021; Du Plessis et al. 2012). For some diurnal species, exposure to hot conditions can be reduced by shifting activity to nocturnal hours, when cooler air temperatures can facilitate the dissipation of metabolic heat; however, this behavioural adjustment is associated with other costs (Hetem et al. 2012; McCain and King 2014; McFarland et al. 2014; Levy et al. 2019). For nocturnal species, activity already occurs during the coolest part of the diel cycle, and hence extremely warm night temperatures could reduce foraging‐related activity, potentially resulting in energy deficits with implications for reproduction and survival.

Here, we measured body acceleration forces as an index of activity and energy expenditure of free‐living adult grey‐headed flying‐foxes ( *Pteropus poliocephalus* ) in summer, including during extreme heat events. These large fruit bats (Pteropodidae) are nocturnal, and roost during the day in tree branches (Churchill 2008), where they are exposed to prevailing environmental conditions. During hot conditions in the roost, flying‐foxes exhibit a sequence of thermoregulatory behaviours, including moving to cooler, shaded microclimates, wing‐fanning to increase air flow and convective heat loss across the skin, profuse salivation, licking of skin at wrists and open‐mouthed panting to increase heat dissipation via evaporative water loss (Bartholomew et al. 1964; Welbergen et al. 2008), and may include flights to ‘belly‐dip’ if open water is present (Markus and Blackshaw 2002).

We tested the hypothesis that extreme heat events cause changes in activity levels that have a substantial impact on the daily energy budget of endothermic animals. Accordingly, we predicted that, during extreme heat events, whole‐body acceleration measurements of flying‐foxes would increase during the daytime resting phase, indicating higher levels of activity and energy expenditure and decrease during the night, suggesting a reduction in foraging compared to mild days. The energy costs of these behavioural responses could accumulate across days of extreme heat, possibly resulting in poor body condition and life‐threatening energy bottlenecks. Deficits in stored body energy could be a compounding cost of extreme heat events, potentially contributing to a reduction in fitness for these bats and other endotherms as extreme heat events intensify under climate change.

## 2 Methods

### 2.1 Animal Capture and Deployment of Transmitters

We used mist nets to catch grey‐headed flying‐foxes at the Botanic Park roosting camp, Adelaide, South Australia (−34.9156° S, 138.6070° E) on 10 and 11 December 2019. Ten adult bats (5 F: 725 g; 5 M: 818 g) were fitted with a solar‐powered GPS and accelerometry logger telemetry units (17 g CREX GPS Logger, Ecotone Telemetry, Poland) under anaesthesia. Telemetry units were attached using neoprene‐lined leather collars (Yabsley et al. 2022), secured by stitches of polyester thread to facilitate breakaway after approximately 12 months. The collar and units were fitted to bats weighing > 550 g and comprised an average of 2.3% of body mass, less than the 3% suggested as a conservative limit for minimising behavioural impacts (Cochran 1980). Similar collar units have been used on this and other *Pteropus* species without any evidence of negative impacts on flight movements (Grady et al. 2025; Welbergen et al. 2020; Todd et al. 2022; Oleksy et al. 2019; Mandl et al. 2022; Yabsley et al. 2022, 2021; Meade et al. 2021; Boardman et al. 2021). Positional fixes (not used here) and acceleration data were received via the GSM cellular network. Air temperature (*T*_*a*_) data at 1‐min resolution were sourced from two Australian Bureau of Meteorology weather stations, 2.9 km west (#023000) and 1.3 km southeast (#023090) of the Botanic Park camp.

### 2.2 Data Handling and Analysis

Data handling and analyses were conducted in R (version 4.1.2; R Core Team 2021) interfaced with RStudio (version 2021.09.0; RStudio Team 2021). For this study, accelerometry data were selected between 13 December 2019 and 14 February 2020 that had a sampling rate of 12 s bursts at 5 Hz data every 15 min from a larger dataset of varied acceleration data sampling regimes, resulting in 8791 bursts across 63 days from a total of nine individuals (4 F, 5 M), as one of the five collared females left the study area and data for this individual were excluded. The *suncalc* function (Grolemund and Wickham 2011) was used to delineate daytime and night periods based on sunrise and sunset times.

We categorised nights into three thermal periods: hot (*n* = 9), mild (*n* = 50) and cool‐front (*n* = 3). Nights were classified as ‘hot’ when the mean nighttime *T*_*a*_ fell within the upper decile, representing the hottest ~10% of nights (mean nightly *T*_*a*_ ≥ 27°C). Nights with mean *T*_*a*_ < 27°C were categorised as ‘mild’. Each daytime was assigned the same category as the night that followed it; thus, the daytime preceding a hot night was classified as ‘hot’. The exceptions were nights associated with visually identified cool‐front weather systems, which caused the sudden ending of extreme heat. These cool nights allowed us to separate the direct effect of current nighttime *T*_*a*_ on activity from any delayed effect of hot daytime conditions (see Table S1 for further details).

Overall dynamic body acceleration (ODBA) was used as a proxy for energy expenditure (Wilson et al. 2006). The relationship between ODBA and oxygen consumption during resting and activity is close‐fitting and linear among a variety of species of mammals and birds (Halsey et al. 2009), providing validation of its use as a relative index of energy expenditure in free‐living animals. We removed static acceleration forces (gravity) by subtracting from each axis the one‐second running mean, summed the absolute values across the *X*, *Y* and *Z* axes, and multiplied by 9.80665 to obtain ODBA in units of m/s^2^ (Brown et al. 2013; Shepard et al. 2008). In addition, we categorised three activity levels (low, moderate and high) using troughs to distinguish modes in frequency distributions of the within‐burst standard deviation applied to the first principal component of a principal components analysis of the three accelerometry axes (Collins et al. 2015), as described in (Yabsley et al. 2022). Large variance over time in the heave axis, and in the first principal component, is typically associated with flight in birds and bats, whereas resting is associated with a relatively small degree of variance (Collins et al. 2015; O'Mara et al. 2019). Hence, we presumed the mode of greatest variance, which we classified as ‘high’ level activity, was most likely to reflect wing flapping during flight. The next highest mode of variance we classified as ‘moderate’ activity, reflecting mostly non‐flight movements of moderate intensity and the mode of least variance we classified as ‘low’ level activity, reflecting bats that were resting (i.e., hanging from a branch) with minimal movement.

To estimate the effect of *T*_*a*_ on ODBA, we fitted linear mixed effects models (LMM) using the function *lmer* of the lme4 package to explain burst‐summed ODBA separately for data recorded over the daytime or night. Initial models included the fixed effects: *T*_*a*_ as a second‐order polynomial, hour of the day, sex, body mass at capture and maximum *T*_*a*_ of the preceding phase as a second‐order polynomial, and individual identity as a random effect on the intercept. To estimate the effects of *T*_*a*_ on the probability of a flying‐fox exhibiting any one of the three levels of activity (low, moderate, or high) relative to the other two levels of activity during the daytime or night, we fitted six different binomial generalised linear mixed effects models (GLMM) using the function *glmer* from the lme4 package. The initial models included as fixed effects: *T*_*a*_ as a second‐order polynomial regression, hour of the day, sex, body mass at capture and maximum *T*_*a*_ for the preceding phase as a second‐order polynomial. Initial models were simplified by the stepwise removal of non‐significant terms, beginning with the highest *p*‐value, to derive a minimum adequate model that included only significant effects. On the GLMM model outputs, we used the function *ggpredict* to estimate the probability of animals being in different activity levels across a range of *T*_*a*_ in 1°C steps.

## 3 Results

**FIGURE 1:**
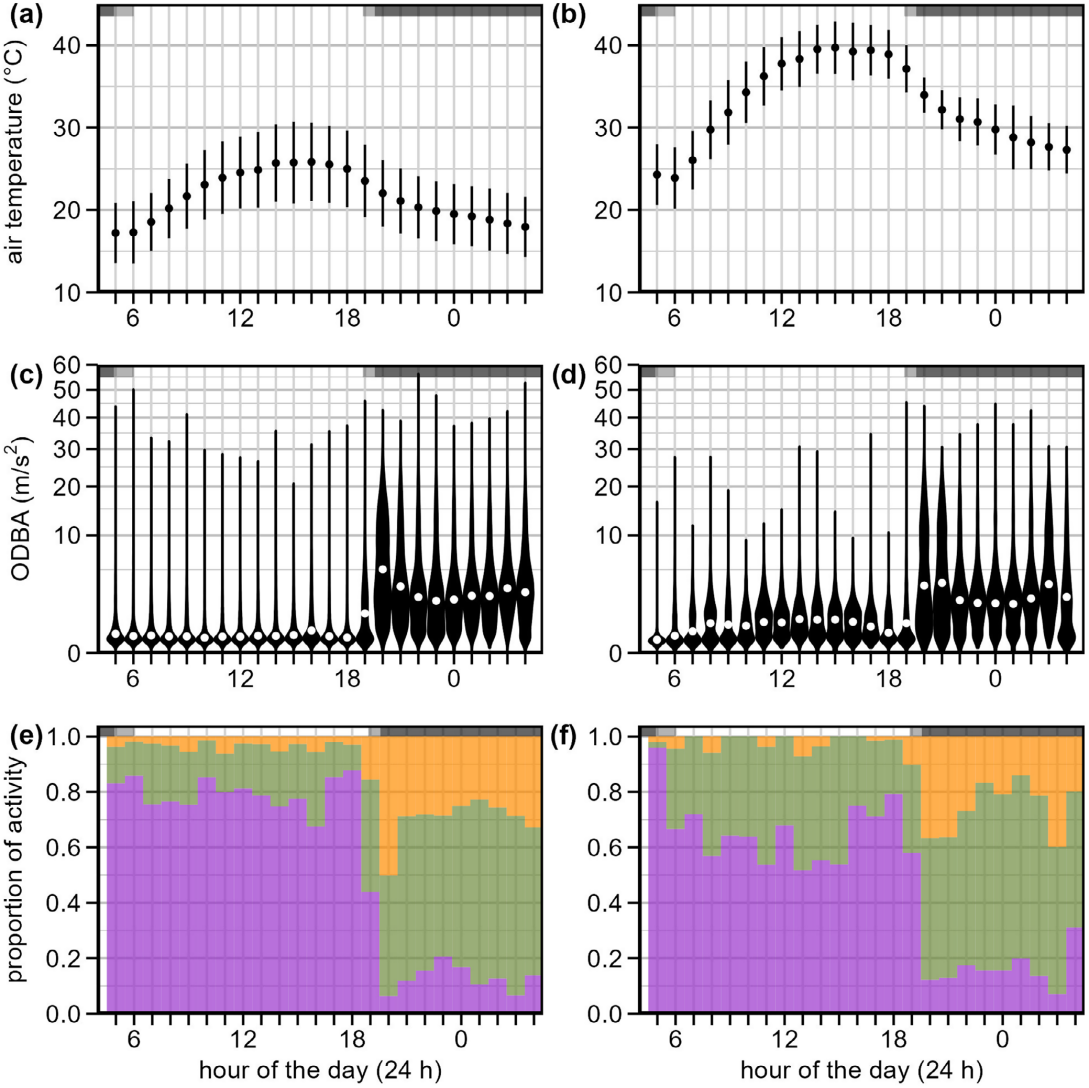
Air temperature (°C), overall dynamic body acceleration (ODBA, m/s^2^) and activity by the grey‐headed flying‐fox, *Pteropus poliocephalus* , (*n* = 9) in summer during relatively mild (a, c, e; *n* = 50) and hot (b, d, f; *n* = 9 periods) periods. Air temperature is shown as the mean ± 1 SD (a, b). The ODBA is shown as hourly frequency distributions, including the mean of individual hourly median values (white dot; c, d). Activity data were derived by tallying counts for each individual by hour and condition, then summing across individuals. For each hour and condition, the proportion of each activity level (low—purple; moderate—green; high—orange; e, f) was calculated as its count divided by the total activity count. Times of sunrise and sunset over the study period are shown by light grey and nighttime by dark grey horizontal shading at the top of each panel.

Hot days were associated with much higher ODBA and hence energy costs during the daytime, and lower ODBA during the early and warmer part of the nighttime, compared to mild days (Figure 1). On hot days, ODBA increased during the first few hours of the morning up until 10 a.m., whereas ODBA remained at low levels on mild days. During hot compared to mild days, the mean of hourly median ODBA values was greater by 219% (i.e., a 3.2‐fold increase) across the 5 h between 11:00 and 16:00 and greater by 140% (2.4‐fold) across the entire daytime. In the first two hours of the night (18:00 to 20:00), the mean of hourly median ODBA was 33% lower on hot compared to mild days. Later during the night, hourly median ODBA values were more similar between hot and mild days. Across all night hours, the mean of hourly median ODBA was 15% lower on hot compared to mild days. These differences in ODBA between hot and mild days were reflected in the classification of levels of activity across the day (Figure 1). A greater proportion of the daytime was classified as moderate activity on hot (0.30) compared to mild (0.17) days. In the first hour after sunset, the proportion of high activity was less on hot (0.37) compared to mild (0.50) days. Over the entire night, high activity was reduced on hot (0.25) compared to mild (0.30) days and low activity was increased on hot (0.17) compared to mild (0.13) days.

**FIGURE 2:**
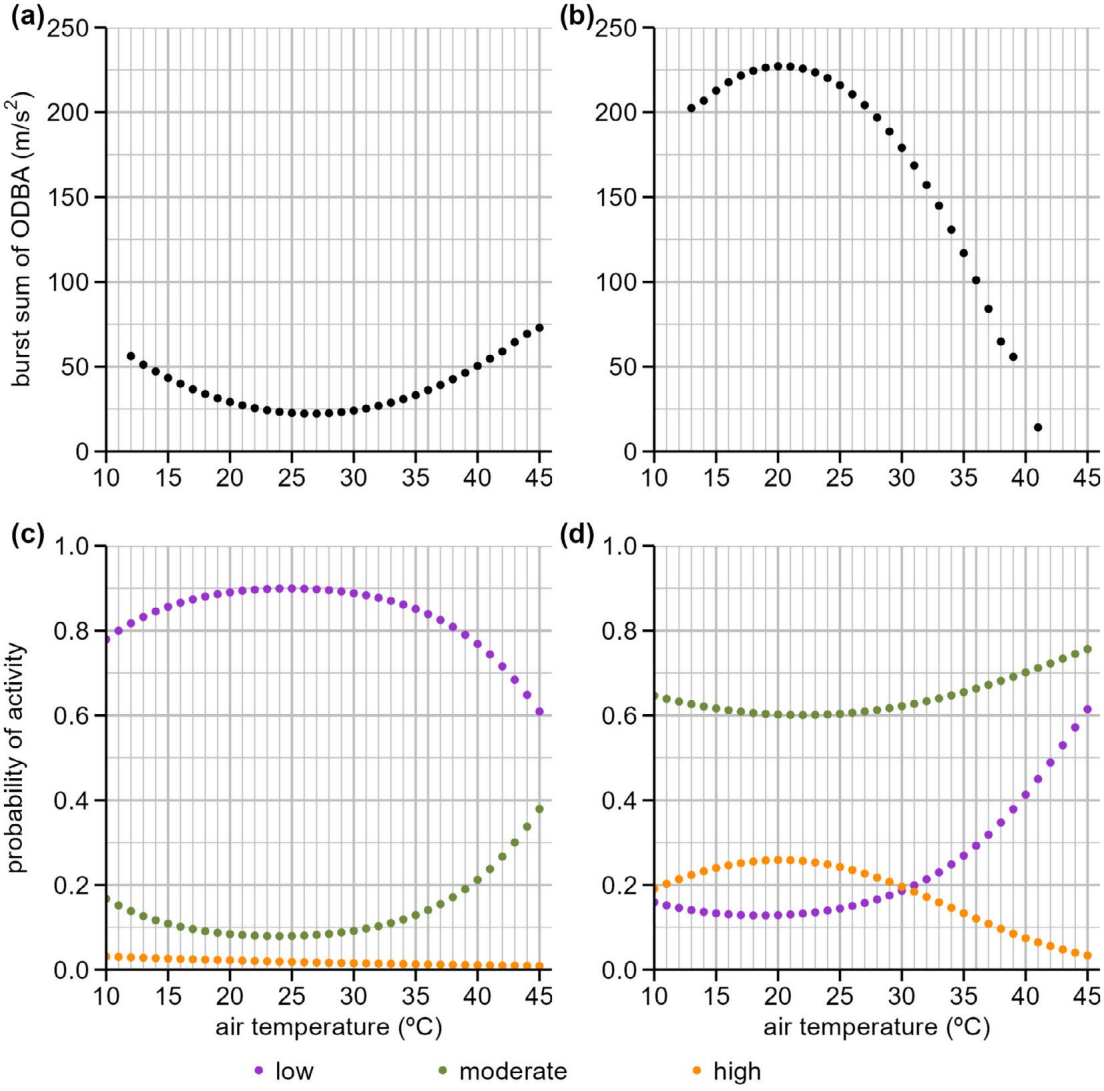
Partial effects plots that describe (a, b) burst‐summed overall dynamic body acceleration (ODBA; m/s^2^) and (c, d) the probability of low, moderate or high levels of activity, as a function of (a, c) daytime and (b, d) nighttime air temperature (°C) for grey‐headed flying‐fox, *Pteropus poliocephalus*, (*n* = 9) during summer. Partial effects were predicted from (a, b) separate linear mixed‐effects models fitted to daytime and nighttime ODBA data (Tables 1 and S2) and (c, d) binomial generalised linear mixed models fitted to each of the six combinations of three activity levels low‐level activity: Purple; moderate‐level: Green; and high‐level and daytime or nighttime (Tables 2 and S3). Partial effects plots show the effect of each variable while constraining the other explanatory variables to their means and include individual identity as a random factor.

The LMM indicated that *T*_*a*_ had strong effects on ODBA that differed between the daytime and nighttime (Figure 2, Table 1). During the daytime, *T*_*a*_ had a convex‐shaped curvilinear effect on ODBA (*p* < 0.001), with ODBA decreasing by ~50% between *T*_*a*_ of 12°C and 21°C and increasing by ~100% between 30°C and 45°C. The final model included significant effects of hour of the day (highest at 19:00; *p* < 0.001) and body mass (positive; *p* = 0.002; Table 1). During the nighttime, *T*_*a*_ had a concave‐shaped curvilinear effect on ODBA (*p* < 0.001), with ODBA beginning to decrease above a *T*_*a*_ of 21°C and decreasing more steeply above a *T*_*a*_ of 25°C. Between *T*_*a*_ of 21°C and 41°C, ODBA at nighttime was predicted to decrease by ~94%. The final model also included significant effects of hour of the day (highest at 20:00; *p* < 0.001; Table 1).

**TABLE 1:** Analysis of deviance from linear mixed‐effects models fitted to explain burst‐summed overall dynamic body acceleration (m/s^2^) of grey‐headed flying‐fox, *Pteropus poliocephalus* , (*n* = 9) during the (a) daytime and (b) nighttime.

(a) Daytime ($R_{\text{marginal}}^{2}$ = 0.04; $R_{\text{conditional}}^{2}$ = 0.04; *n* = 9; *N* = 4200)			
Fixed effects	𝜒2	df	*p*
(intercept)	81.5	1	< 0.001
*T*_*a*_	49.6	1	< 0.001
$T_{a}^{2}$	56.4	1	< 0.001
hour	124.2	14	< 0.001
mass	9.6	1	0.002

Random effects	Variance	SD
Id	785.7	280.0
Residual	51562.0	1227.1
Removed fixed‐effects: sex, maximum *T*_*a*_ for the preceding phase fitted as a second order polynomial		

(b) Nighttime ($R_{\text{marginal}}^{2}$ = 0.09; $R_{\text{conditional}}^{2}$ = 0.10; *n* = 9; *N* = 4530)			
Fixed effects	𝜒^2^	df	*p*
(intercept)	0.1	1	0.781
*T*_*a*_	18.1	1	< 0.001
$T_{a}^{2}$	24.7	1	< 0.001
Hour	400.4	10	< 0.001

Random effects	Variance	SD
Id	978.1	31.3
Residual	51591.8	227.1
Removed fixed‐effects: sex, mass, maximum *T*_*a*_ for the preceding phase fitted as a second order polynomial		

*Note:* The estimates for the fixed effects are presented in Table S3.

We found that *T*_*a*_ had strong effects on the probability of a bat exhibiting a given level of activity (GLMM, Figure 2, Table 2). During the daytime, increasing *T*_*a*_ between 30°C and 45°C was predicted to decrease the probability of low activity from 90% to 60% and increase the probability of moderate activity from 18% to 38% (both *p* < 0.001), whereas *T*_*a*_ did not significantly influence high activity (*p* = 0.984). At nighttime, *T*_*a*_ increasing above approximately 21°C reduced the probability of high activity (*p* < 0.001), which was predicted to decrease from 27% at 21°C to 13% at 35°C and 3% at 45°C and increased the probability of low activity (*p* = 0.007), which was predicted to increase from 15% at 21°C to 28% at 35°C and 62% at 45°C, whereas *T*_*a*_ did not significantly influence moderate activity (*p* = 0.140). Hour of the day, sex and body mass were also significant fixed effects in some of these GLMMs (Table 2).

**TABLE 2:** Analysis of deviance (Type III Wald chi‐square tests) and random effects from six binomial generalised linear mixed models fitted by maximum likelihood to explain the probability of a grey‐headed flying‐fox, *Pteropus poliocephalus* , exhibiting low‐, moderate‐ or high‐levels of activity during the (a) daytime (*N* = 4200) or (b) the nighttime (*N* = 4530).

(a) Daytime	Low activity			Moderate activity			High activity		
Fixed effects	*𝝌* ^2^	df	*p*	*𝝌* ^2^	df	*p*	*𝝌* ^2^	df	*p*
(intercept)	0.8	1	0.358	0.1	1	0.895	1.2	1	0.283
*T*_*a*_	40.6	1	< 0.001	36.6	1	< 0.001	0.1	1	0.796
$T_{a}^{2}$	54.5	1	< 0.001	53.1	1	< 0.001	0.1	1	0.984
Hour	105.9	14	< 0.001	78.5	14	< 0.001	42.6	14	< 0.001
Sex	4.6	1	0.033	8.5	1	0.004	6.2	1	0.013
Mass	—	—	—	—	—	—	3.6	1	0.059

Random effects	Var.	SD	Var.	SD	Var.	SD
Id	0.01	0.08	0.01	0.08	0.00	0.00

*R* ^2^ values	Marg.	Cond.	Marg.	Cond.	Marg.	Cond.
Theoretical	0.08	0.08	0.09	0.09	0.12	0.12
Delta	0.05	0.05	0.05	0.05	0.02	0.02

(b) Nighttime	Low activity			Moderate activity			High activity		
Fixed effects	*𝝌* ^2^	df	*p*	*𝝌* ^2^	df	*p*	*𝝌* ^2^	df	*p*
(intercept)	1.0	1	0.315	4.3	1	0.038	20.2	1	< 0.001
*T*_*a*_	4.3	1	0.038	1.9	1	0.172	9.3	1	0.002
$T_{a}^{2}$	7.2	1	0.007	2.2	1	0.140	12.7	1	< 0.001
Hour	102.4	10	< 0.001	85.4	10	< 0.001	141.4	10	< 0.001

Random effects	Var.	SD	Var.	SD	Var.	SD
Id	0.14	0.37	0.07	0.25	0.04	0.21

*R* ^2^ values	Marg.	Cond.	Marg.	Cond.	Marg.	Cond.
Theoretical	0.07	0.11	0.02	0.04	0.29	0.29
Delta	0.03	0.04	0.02	0.04	0.21	0.22

*Note:* The estimates for the fixed effects are presented in Table S3.

## 4 Discussion

Our study used acceleration to quantify activity and derive ODBA, an index of energy expenditure (Wilson et al. 2006; Halsey et al. 2009), in free‐living grey‐headed flying‐foxes over natural summer conditions that included extremely hot weather events. During the daytime resting period, hot conditions increased moderate‐level activity associated with thermoregulatory behaviours (e.g., wing‐fanning, licking and panting), resulting in substantially higher ODBA compared to relatively mild summer conditions. Whereas, during the nighttime, even moderately hot air temperatures decreased high‐level activity indicative of flight. Although we measured only nine adult individuals, the magnitude and consistency of the responses suggest relevance beyond the sampled cohort. Collectively, these shifts in activity during extreme heat events represent a substantial energetic cost that is likely to negatively impact an individual's body energy reserves. Depending on the duration and frequency of extreme heat events, such energy deficits could have longer‐term consequences through reduced survival or reproductive success, particularly in already stressed or resource‐limited populations. As extreme heat events intensify and become more frequent under anthropogenic climate change, the energetic costs of behavioural responses to extreme heat are likely to represent an important mechanism driving negative impacts on bat populations and other exposed wildlife.

Extreme daytime heat increased rest‐phase activity in flying‐foxes, as revealed by accelerometry, resulting in a substantially elevated ODBA under hot conditions. On hot days, the frequency distribution of daytime ODBA shifted to overlap markedly with that of the nocturnal active phase, with median hourly values across most of the daytime exceeding that of mild days by more than threefold. The increase in ODBA at air temperatures above ~35°C accords with the onset of more vigorous thermoregulatory behaviours, such as wing fanning (Bartholomew et al. 1964) and with a steeper controlled increase in body temperature in grey‐headed flying‐foxes at this same field site (Walker et al. 2025). Activity and ODBA increased most strongly above 40°C, when air temperature begins to exceed body temperature in this species (Walker et al. 2025), making evaporative cooling the only means for heat dissipation. Although hot conditions at this study site are typically associated with relatively dry continental air, the negative effect of humidity on evaporative cooling efficiency (Freeman et al. 2024) suggests that energy costs could be even greater in more humid locations, potentially increasing the ecological consequences of heat‐related energy deficits.

In addition to an energetic cost, the activity associated with hot conditions would result in a reduction in time spent sleeping during the day. Reductions in body temperature of grey‐headed flying‐foxes during the early morning and late afternoon (Turbill et al. 2024; Walker et al. 2025) suggest that these are the primary times of the day when bats are likely to sleep. However, during extreme heat events, air temperature warms quickly during the early morning to levels associated with active thermoregulation (i.e., > 35°C) and remains high until after sunset, presumably restricting daytime sleep to a narrow window around dawn. Hot summer conditions have been reported to restrict sleep in the Indian flying‐fox, *Pteropus giganteus* (Roy et al. 2020) and Wahlberg's epauletted fruit bat, *Epomophorus wahlbergi* (Downs et al. 2015). A sleep deficit negatively affects physiological homeostasis, and recovery can also impinge on the functions of wakefulness (Siegel 2005). The detrimental effects of activity during extreme heat events on physiological functions of sleep, which include energy conservation, are likely to accumulate during prolonged weather events, potentially intensifying negative effects and extending a recovery period.

Hot temperatures reduce high‐level activity (which we assume was primarily flight) at nighttime, likely because of a reduced capacity to dissipate metabolic heat. Combined with negative effects of hot conditions on nectar availability (Alquichire‐Rojas et al. 2024), this could reduce rates of energy intake during extreme heat events. Relatively little research has examined how nocturnally active endothermic animals adjust activity in response to increasingly hot conditions, which is particularly important for bats given the high metabolic rates of flight compared to terrestrial locomotion (Thomas 1975). Reduced activity is a universal response of endothermic animals exposed to hot conditions during their active phase (Hetem et al. 2012; McCain and King 2014; McFarland et al. 2014; Levy et al. 2019). In the grey‐headed flying‐fox, flight at intermediate speeds in the laboratory resulted in hyperthermia at an air temperature as low as 25°C (Carpenter 1985). Consistent with this, as nighttime air temperature increased from 25°C to 35°C, ODBA was reduced by approximately 50%. Over this temperature range, the probability of high‐level activity was also halved, suggesting that bats spent less time flying on hot nights. In contrast, on cool nights following hot days, bats showed greater rates of high‐level activity than on hot nights (Figure S1, Table S1), supporting an interpretation that reduced high‐level activity was associated with high nighttime air temperatures rather than delayed effects of daytime heat. Even moderately high nighttime air temperatures (e.g., > 25°C) therefore appear sufficient to reduce flight activity, potentially constraining foraging opportunities and energy intake during heat events.

Our results support the hypothesis that extreme heat is associated with energetic bottlenecks arising from the combination of much higher rest‐phase energy expenditure caused by thermoregulatory behaviours and reduced activity because of limits to dissipation of metabolic heat and hence a reduced rate of food intake. Such energy deficits may contribute directly to mortality during a prolonged heat event, potentially manifesting as lethal hyperthermia when individuals can no longer sustain the energetic cost of active thermoregulatory behaviours. Furthermore, greater daytime energy use and limited nighttime opportunities to replenish energy reserves increase the risk of dehydration and starvation during subsequent days. Mortality risk is likely to be compounded by negative effects of heat on nectar availability (Alquichire‐Rojas et al. 2024) and by cumulative declines in body condition from repeated exposure to extreme heat events during a summer. Over longer timeframes, heat‐linked reductions in body condition could reduce survival and reproductive success, thereby suppressing population growth in exposed species. As extreme heat events intensify under climate change, the combined physiological and ecological consequences may increasingly threaten population persistence and the maintenance of ecosystem functions in heat‐exposed species.

## Author Contributions

**Melissa J. Walker:** conceptualization (equal), formal analysis (equal), investigation (equal), validation (equal), writing – original draft (equal). **Justin A. Welbergen:** conceptualization (equal), funding acquisition (equal), supervision (equal), writing – review and editing (equal). **Jessica Meade:** conceptualization (equal), supervision (equal), writing – review and editing (equal). **Wayne S. J. Boardman:** methodology (equal), writing – review and editing (equal). **John M. Martin:** methodology (equal), writing – review and editing (equal). **Christopher Turbill:** conceptualization (equal), funding acquisition (equal), supervision (equal), writing – original draft (equal).

## Funding

This project received funding from an Australian Research Council Discovery Grant (DP170104272) awarded to Welbergen and Turbill, and from the Hawkesbury Institute for the Environment and WSU Candidature Project Funding awarded to Walker. The purchase of GPS collars was facilitated by Jason Van Weenen and supported by Natural Resources Adelaide and Mt. Lofty Ranges.

## Ethics Statement

Study and research permits were issued by the Department of Environment, Water and Natural Resources, Government of South Australia (M26735‐3) and the Western Sydney University Animal Care and Ethics Committee (A12217).

## Conflicts of Interest

The authors declare no conflicts of interest.

## Supporting information


**Figure S1:** ece373408‐sup‐0001‐Supinfo.docx.

## Data Availability

All the required data are uploaded as Supporting Information. Air temperature data (air_temp) have been intentionally excluded, as these data were sourced from the Australian Bureau of Meteorology and are subject to licensing restrictions.
