# Supplementary material for: Energetic Costs of Extreme Heat: High Temperatures Elevate Daytime Activity and Suppress Nighttime Foraging in Flying‐Foxes
Source: Ecol Evol. 2026 Apr 8;16(4):e73408. doi: 10.1002/ece3.73408 (PMC13062654; doi:10.1002/ece3.73408)
Supplement: Supplementary file 1 — Figure S1: ece373408‐sup‐0001‐Supinfo.docx. [file ECE3-16-e73408-s001.docx]

**Title:** Energetic costs of extreme heat: high temperatures elevate daytime activity and suppress nighttime foraging in flying-foxes

**Supplementary material**


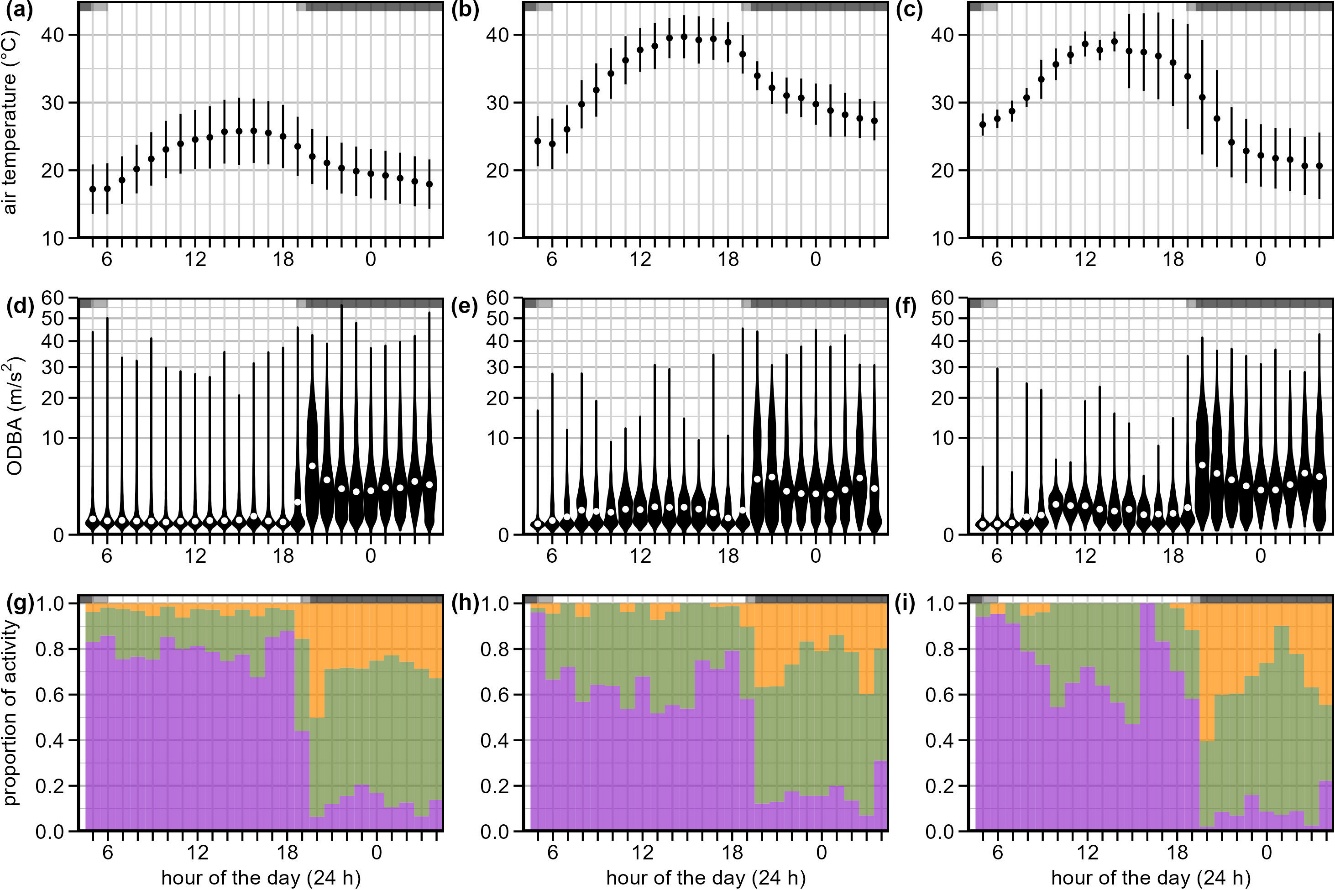


Figure S1. Overall dynamic body acceleration (ODBA, m/s^2^) and activity by the grey-headed flying-fox, *Pteropus poliocephalus*, (*n* = 9) in summer during relatively mild (a, d, g; *n* = 50), hot (b, e, h; *n* = 9 periods), and cool-front periods (c, f, i; *n* = 3 periods). Air temperature (°C) is shown as the mean ± 1 SD (a, b, c). The ODBA is shown as hourly frequency distributions, including the mean of individual hourly median values (white dot; d, e, f). Activity data were derived by tallying counts for each individual by hour and condition, then summing across individuals. For each hour and condition, the proportion of each activity level (low - purple; moderate - green; high – orange; g, h, i) was calculated as its count divided by the total activity count. Times of sunrise and sunset over the study period are shown by light grey and night by dark grey horizontal shading at the top of each panel.

Table S1. Air temperature (T_a_, °C) metrics for three thermal periods: mild (*n* = 50), hot (*n* = 9, 8 during a heatwave), and cool-front (*n* = 3). Nights (a) with a mean T_a_ < 27 °C were classified as mild and ≥ 27 °C as hot. The 27 °C threshold corresponds to the upper decile of mean nighttime T_a_, capturing the hottest ~10 % of nights. Days (b) preceding each night were assigned the same category, except for the visually identified cool-fronts.

| phase | night | | | day | | |
| --- | --- | --- | --- | --- | --- | --- |
| thermal period | mild | hot | cool-front | mild | hot | cool-front |
| abs. T_a, min_ | 13.3 | 23.7 | 15.3 | 11.9 | 15.8 | 23.9 |
| mean T_a, min_ (±1SD) | 17.5 ± 3.4 | 26.4 ± 3.0 | 20.1 ± 4.4 | 16.5 ± 3.0 | 23.3 ± 3.6 | 25.5 ± 1.4 |
| mean T_a, mean_ (±1SD) | 19.7 ± 3.6 | 30.1 ± 2.7 | 23.8 ± 5.1 | 22.7 ± 3.8 | 34.5 ± 3.2 | 33.9 ± 2.4 |
| mean T_a, max_ (±1SD) | 23.0 ± 4.4 | 36.2 ± 2.6 | 33.3 ± 8.1 | 27.0 ± 4.5 | 40.6 ± 3.1 | 41.0 ± 0.5 |
| abs. T_a, max_ | 31.2 | 40.7 | 38.5 | 35.1 | 44.6 | 41.7 |

Table S2. Fixed effects table a linear mixed-effects model to explain burst summed overall dynamic body acceleration (m/s^2^) of grey-headed flying-foxes, *Pteropus poliocephalus*, during the (a) day and (b) the night. A Plot of the model-estimated partial effect of air temperature (T_a_, °C) is presented in figure 2 and the analysis of deviance is presented in table 1.

| **(a) day**  (R^2^_marginal_ = 0.04; R^2^_conditional_= 0.04; *n* = 9; *N* = 4200) | | | | | |
| --- | --- | --- | --- | --- | --- |
| fixed effect | est. | SE | DF | *t* | *p* |
| (intercept) | 160.0 | 17.7 | 78.2 | 9.0 | <0.001 |
| T_a_ | -8.3 | 1.2 | 3770.4 | -7.0 | <0.001 |
| T_a_ ^2^ | 0.2 | 0.0 | 3327.7 | 7.5 | <0.001 |
| hour - 6 | 2.9 | 7.4 | 4179.7 | 0.4 | 0.694 |
| hour - 7 | 11.9 | 7.2 | 4179.0 | 1.7 | 0.097 |
| hour - 8 | 21.3 | 7.4 | 4181.9 | 2.9 | 0.004 |
| hour - 9 | 17.6 | 7.0 | 4180.6 | 2.5 | 0.012 |
| hour - 10 | 8.3 | 7.2 | 4181.8 | 1.2 | 0.247 |
| hour - 11 | 24.1 | 7.3 | 4179.2 | 3.3 | 0.001 |
| hour - 12 | 17.9 | 7.4 | 4181.4 | 2.4 | 0.015 |
| hour - 13 | 18.8 | 7.2 | 4181.3 | 2.6 | 0.009 |
| hour - 14 | 25.6 | 7.2 | 4177.7 | 3.6 | 0.000 |
| hour - 15 | 14.9 | 7.5 | 4179.1 | 2.0 | 0.047 |
| hour - 16 | 30.1 | 9.3 | 4180.4 | 3.3 | 0.001 |
| hour - 17 | 12.2 | 7.2 | 4179.4 | 1.7 | 0.094 |
| hour - 18 | 9.7 | 6.6 | 4166.5 | 1.5 | 0.146 |
| hour - 19 | 59.2 | 7.0 | 4181.3 | 8.4 | <0.001 |
| mass | 0.0 | 0.0 | 5.0 | -3.1 | 0.027 |
| **(b) night**  (R^2^_marginal_ = 0.09; R^2^_conditional_= 0.10; *n* = 9; *N* = 4530) | | | | | |
| fixed effect | est. | SE | DF | *t* | *p* |
| (intercept) | 16.0 | 57.5 | 2379.7 | 0.3 | 0.781 |
| T_a_ | 20.8 | 4.9 | 4515.1 | 4.3 | <0.001 |
| T_a_ ^2^ | -0.5 | 0.1 | 4515.1 | -5.0 | <0.001 |
| hour - 1 | 7.9 | 14.4 | 4509.1 | 0.5 | 0.584 |
| hour - 2 | 35.1 | 14.3 | 4509.3 | 2.5 | 0.014 |
| hour - 3 | 89.4 | 14.4 | 4510.4 | 6.2 | <0.001 |
| hour - 4 | 55.7 | 14.8 | 4509.7 | 3.8 | 0.000 |
| hour - 5 | -138.5 | 54.7 | 4512.2 | -2.5 | 0.011 |
| hour - 19 | 2.9 | 21.4 | 4510.7 | 0.1 | 0.893 |
| hour - 20 | 222.6 | 14.5 | 4509.9 | 15.4 | <0.001 |
| hour - 21 | 121.2 | 15.6 | 4508.9 | 7.8 | <0.001 |
| hour - 22 | 45.2 | 14.3 | 4508.7 | 3.2 | 0.002 |
| hour - 23 | 13.7 | 14.3 | 4510.3 | 1.0 | 0.337 |

Table S3. Fixed effects from six generalised linear mixed models fitted by maximum likelihood to explain the probability of a grey-headed flying-fox, *Pteropus poliocephalus*, exhibiting low, moderate, and high activity during the (a) day (*n* = 9, *N* = 4200;) or (b) the night (*n* = 9, *N* = 4530). A Plot of the model-estimated partial effect of air temperature (T_a_, °C) is presented in figure 2 and the analysis of deviance is presented in table 2.

| **(a) day** | low activity | | | | moderate activity | | | | high activity | | | |
| --- | --- | --- | --- | --- | --- | --- | --- | --- | --- | --- | --- | --- |
| fixed effect | est. | SE | *z* | *p* | est. | SE | *z* | *p* | est. | SE | *z* | *p* |
| (intercept) | -0.4 | 0.5 | -0.9 | 0.358 | 0.1 | 0.5 | 0.1 | 0.895 | -1.8 | 1.7 | -1.1 | 0.283 |
| T_a_ | 0.2 | 0.0 | 6.4 | <0.001 | -0.2 | 0.0 | -6.1 | <0.001 | 0.0 | 0.1 | -0.3 | 0.796 |
| T_a_ ^2^ | 0.0 | 0.0 | -7.4 | <0.001 | 0.0 | 0.0 | 7.3 | <0.001 | 0.0 | 0.0 | 0.0 | 0.984 |
| hour - 6 | -0.4 | 0.3 | -1.5 | 0.133 | 0.5 | 0.3 | 1.8 | 0.077 | -0.1 | 0.6 | -0.2 | 0.823 |
| hour - 7 | -0.9 | 0.3 | -3.7 | <0.001 | 1.2 | 0.3 | 4.2 | <0.001 | -0.4 | 0.6 | -0.7 | 0.471 |
| hour - 8 | -1.1 | 0.3 | -4.5 | <0.001 | 1.2 | 0.3 | 4.4 | <0.001 | 0.5 | 0.5 | 0.9 | 0.380 |
| hour - 9 | -1.1 | 0.2 | -4.5 | <0.001 | 1.2 | 0.3 | 4.2 | <0.001 | 0.6 | 0.5 | 1.2 | 0.236 |
| hour - 10 | -0.8 | 0.3 | -3.0 | 0.003 | 1.0 | 0.3 | 3.4 | 0.001 | -0.9 | 0.7 | -1.2 | 0.230 |
| hour - 11 | -1.0 | 0.3 | -3.9 | <0.001 | 1.0 | 0.3 | 3.4 | 0.001 | 0.8 | 0.5 | 1.7 | 0.087 |
| hour - 12 | -0.7 | 0.3 | -2.6 | 0.008 | 0.8 | 0.3 | 2.9 | 0.003 | -0.4 | 0.7 | -0.6 | 0.525 |
| hour - 13 | -1.0 | 0.3 | -4.1 | <0.001 | 1.1 | 0.3 | 3.9 | <0.001 | 0.4 | 0.5 | 0.8 | 0.438 |
| hour - 14 | -1.1 | 0.3 | -4.4 | <0.001 | 1.1 | 0.3 | 4.0 | <0.001 | 0.8 | 0.5 | 1.6 | 0.114 |
| hour - 15 | -1.0 | 0.3 | -4.0 | <0.001 | 1.2 | 0.3 | 4.1 | <0.001 | -0.1 | 0.6 | -0.2 | 0.864 |
| hour - 16 | -0.8 | 0.3 | -2.6 | 0.010 | 0.8 | 0.3 | 2.4 | 0.016 | 0.5 | 0.7 | 0.8 | 0.419 |
| hour - 17 | -0.4 | 0.3 | -1.4 | 0.153 | 0.5 | 0.3 | 1.7 | 0.097 | -0.3 | 0.6 | -0.4 | 0.682 |
| hour - 18 | -0.3 | 0.3 | -1.1 | 0.279 | 0.3 | 0.3 | 1.1 | 0.282 | 0.1 | 0.5 | 0.3 | 0.797 |
| hour - 19 | -1.6 | 0.2 | -6.5 | <0.001 | 1.4 | 0.3 | 5.3 | <0.001 | 1.3 | 0.5 | 2.9 | 0.003 |
| sex (m) | 0.2 | 0.1 | 2.1 | 0.033 | -0.3 | 0.1 | -2.9 | 0.004 | 0.5 | 0.2 | 2.5 | 0.013 |
| mass | – | – | – | – | – | – | – | – | 0.0 | 0.0 | -1.9 | 0.059 |
| **(b) night** | low activity | | | | moderate activity | | | | high activity | | | |
| fixed effect | est. | SE | *z* | *p* | est. | SE | *z* | *p* | est. | SE | *z* | *p* |
| (intercept) | -0.7 | 0.7 | -1.0 | 0.315 | 1.1 | 0.5 | 2.1 | 0.038 | -2.6 | 0.6 | -4.5 | 0.000 |
| T_a_ | -0.1 | 0.1 | -2.1 | 0.038 | -0.1 | 0.0 | -1.4 | 0.172 | 0.2 | 0.0 | 3.1 | 0.002 |
| T_a_ ^2^ | 0.0 | 0.0 | 2.7 | 0.007 | 0.0 | 0.0 | 1.5 | 0.140 | 0.0 | 0.0 | -3.6 | <0.001 |
| hour - 1 | -0.3 | 0.2 | -1.6 | 0.105 | 0.3 | 0.1 | 2.6 | 0.011 | -0.2 | 0.2 | -1.6 | 0.114 |
| hour - 2 | -0.2 | 0.2 | -1.3 | 0.179 | 0.1 | 0.1 | 0.9 | 0.353 | 0.0 | 0.1 | 0.1 | 0.927 |
| hour - 3 | -1.0 | 0.2 | -4.5 | <0.001 | 0.1 | 0.1 | 0.6 | 0.539 | 0.4 | 0.1 | 2.6 | 0.010 |
| hour - 4 | 0.2 | 0.2 | 1.0 | 0.333 | -0.4 | 0.1 | -3.0 | 0.003 | 0.4 | 0.1 | 2.6 | 0.010 |
| hour - 5 | 2.5 | 0.5 | 4.8 | 0.000 | -1.2 | 0.5 | -2.3 | 0.019 | -16.9 | 81.0 | -0.2 | 0.834 |
| hour - 19 | -0.2 | 0.3 | -0.9 | 0.381 | -0.1 | 0.2 | -0.8 | 0.436 | 0.3 | 0.2 | 1.2 | 0.219 |
| hour - 20 | -1.1 | 0.2 | -5.1 | <0.001 | -0.7 | 0.1 | -5.1 | <0.001 | 1.2 | 0.1 | 8.6 | <0.001 |
| hour - 21 | -0.4 | 0.2 | -2.1 | 0.039 | -0.2 | 0.1 | -1.1 | 0.281 | 0.4 | 0.2 | 2.7 | 0.006 |
| hour - 22 | -0.1 | 0.2 | -0.4 | 0.723 | -0.2 | 0.1 | -1.3 | 0.205 | 0.2 | 0.1 | 1.7 | 0.094 |
| hour - 23 | 0.3 | 0.2 | 1.5 | 0.123 | -0.2 | 0.1 | -1.8 | 0.066 | 0.1 | 0.1 | 0.8 | 0.452 |
